# The use of plant community attributes to detect habitat quality in coastal environments

**DOI:** 10.1093/aobpla/plw040

**Published:** 2016-07-11

**Authors:** Silvia Del Vecchio, Antonio Slaviero, Edy Fantinato, Gabriella Buffa

**Affiliations:** Centre for Estuarine and Marine Studies, DAIS, University Ca’ Foscari of Venice, Castello 2737b, 30122 Venice, Italy

**Keywords:** Coastal dune ecosystems, community structure, community level variables, habitat monitoring, species ecological groups

## Abstract

The monitoring of biodiversity has mainly focused on the species level. However, researchers and land managers are making increasing use of complementary assessment tools that address higher levels of biological organization, i.e. communities, habitats and ecosystems. Recently, a variety of frameworks have been proposed for assessing the conservation status of communities or ecosystems. Among the various criteria proposed, all the protocols suggest considering (i) spatial aspects (range and area), and (ii) qualitative aspects of specific structures and functions. However, changes to ecological function are difficult to quantify and many protocols end up by using qualitative criteria. The aim of this work was to test the efficacy of some plant community attributes for the detection of vegetation quality in sand dune plant communities. We chose plant community attributes that either help to distinguish a habitat from others (diagnostic components) or play a significant role in habitat function and persistence over time. We used a diachronic approach by contrasting up-to-date vegetation data with data from previous studies carried out within the same areas. Changes in species composition were detected through detrended correspondence analyses (detrended correspondence analyses), Multi-Response Permutation Procedures and Indicator Species Analysis, while structural changes were analyzed by comparing species richness, total species cover, ecological groups of species and growth forms through null models. Ecological groups such as native focal species and aliens, and growth forms proved their efficacy in discriminating between habitat types and in describing their changes over time. The approach used in this study may provide an instrument for the assessment of plant community quality that can be applied to other coastal ecosystems.

## Abstract (precis)

To detect changes in coastal ecosystems, we evaluated the variation over time in some vegetation features, such as species composition and structure (species richness, cover, growth forms). We found that ecological groups of species such as native focal species (species that provide essential ecological functions) and aliens (species that spread outside their natural distribution), and growth forms proved their efficacy in discriminating between habitat types and in describing their changes over time. The approach used in the current study may provide an instrument for the assessment of plant community quality that can be applied to other coastal ecosystems.

## Introduction

Globally, countries are experiencing the degradation and loss of coastal habitats (Curr *et al.* 2000; Defeo *et al.* 2009; EEA 2009; Feagin *et al.* 2005). Economic progress, burgeoning human populations as well as the growing demand for coast-bound tourism opportunities have increased pressures on sandy beaches (Dugan and Hubbard 2010) and coastal sandy ecosystems are currently identified as one of the most threatened ecosystems prone to biodiversity loss (EEA 2009). Given the growing empirical and theoretical evidence that ecosystem functions and services are linked to biodiversity (Cardinale *et al.* 2012; Hooper *et al.* 2012), it can be expected that the loss of species and habitats will affect pivotal ecosystem functions which form the basis of the distinctive ecological services provided by coastal ecosystems, such as erosion and salt spray control, storm buffering, water filtration, nutrient recycling.

Hitherto, the monitoring of biodiversity has mainly focused on the species level, with species-level assessments of extinction risk having been used to set priorities for conservation (Mace *et al.* 2008; Margules and Pressey 2000). However, researchers and land managers are making increasing use of complementary assessment tools that address higher levels of biological organization, i.e. ecological communities, habitats and ecosystems (Keith 2009; Keith *et al.* 2013; Nicholson *et al.* 2009). Indeed, plant communities or vegetation types represent a key approach for biodiversity conservation above the species level and have been increasingly used as crucial units for inventory, planning and monitoring as they are good indicators of overall biodiversity. Moreover, they are able to provide information about underlying abiotic components and to document individual species’ ecological requirements (Benavent-González *et al.* 2014; Peet and Roberts 2012).

In Europe, vegetation types have achieved a legal status as they are used to define endangered habitats according to the Habitats Directive 92/43 (EEC 1992), which aims to ensure biodiversity by conserving natural habitats and wild fauna and flora in the territory of the Member States. The Directive requires governments to designate and protect a national network of sites (the Natura 2000 network) and to provide monitoring, management and all appropriate measures to maintain, or restore, habitats at a ‘Favourable Conservation Status’ (FCS). The concept of FCS is central to the EC Habitats Directive and means that a habitat’s natural range and area are stable or increasing and the species structure and functions which are necessary for its long term maintenance exist and are likely to continue to exist for the foreseeable future. Finally, the populations of its typical species are stable and self-maintaining (Jones 2002). The FCS issue is particularly challenging for sandy coastal ecosystems, where plant communities have long proved to be critical elements in relation to the morphology and dynamics of the entire dune system (Stallins 2005).

Recently, a variety of frameworks has been proposed for assessing the conservation status of habitats or ecosystems (e.g. Evans and Arvela 2011; Keith *et al.* 2013; New South Wales Scientific Committee 2012; Nicholson *et al.* 2009; Rodríguez *et al.* 2011, 2012; Walker *et al.* 2006). Although based on different approaches and different scales, all the protocols suggest considering both spatial (range and area, and rate of decline in distribution) and qualitative aspects. Stemming from the protocol for species risk assessment (Mace *et al.* 2008), spatial criteria make direct reference to declining population and small population paradigms. Qualitative aspects refer to specific structures (physical components) and functions (ecological processes) necessary for the long-term maintenance of the community, and relate to properties that involve manifold species and interactions between species and between species and their environment (Keith 2009).

Although reduction in distribution is relatively easily detected, the recognition of discrete thresholds and endpoints of the structural or functional decline of a vegetation type is difficult (Nicholson *et al.* 2009), since a vegetation type can undergo a slow decline that leads to transformation into a new one, with a different species composition and with weakened or altered functions (Hobbs *et al.* 2006). However, before extinction occurs, proxies can be used to evaluate changes to ecological functions. Such proxies can be related to community structure and species composition, e.g. focal, keystone or dominant species (e.g. Benson 2006; Keith *et al.* 2013; Lindgaard and Henriksen 2011), functions of component species, disruption of ecological processes, such as disturbance regimes, or alien invasion (e.g. Keith *et al.* 2013; Lindgaard and Henriksen 2011; New South Wales Scientific Committee 2012).

In this context, the concept of ‘diagnostic species composition’, a kind of ‘reference state’ (Reynoldson and Wright 2000), becomes central. Hence, such plant community attributes as the presence, abundance or dominance of key species, i.e. structural or functional unique elements, or groups of species that share ecological requirements and features of importance for determining habitat structure and composition (i.e. ecological groups or functional groups) may be used for a practical analysis of plant community quality (Cardinale *et al.* 2012; Keith *et al.* 2013). Many authors (Keith 2009; Keith *et al.* 2013; Nicholson *et al.* 2009) agree that when applied effectively, ecological communities or habitats can be powerful tools for achieving cost-effective outputs in land-use planning and biodiversity conservation. However, their recent application in different contexts has evidenced some critical aspects and it remains difficult to quantify the degradation of communities and incorporate it in assessment protocols. One solution could be to develop consistent and transparent sub-criteria for specific types of degradation (Nicholson *et al.* 2009), or for specific habitat types (Gigante *et al.* 2015). Such an approach to assessing habitat quality might help to implement strategic management plans underpinned by a sound theoretical background.

On this basis, the aim of this study was to test the efficacy of some plant community attributes for the detection of habitat quality in coastal environments. We analyzed both plant species composition and structure, considering variables that either help to distinguish a habitat from others (diagnostic components) or play a significant role in habitat function and persistence over time. To test the efficacy of chosen attributes in disclosing changes over time we used a diachronic approach by contrasting up-to-date vegetation data with data from previous studies carried out using the same field protocol and survey method and at the same sites.

## Methods

### Study area

**Figure 1.**
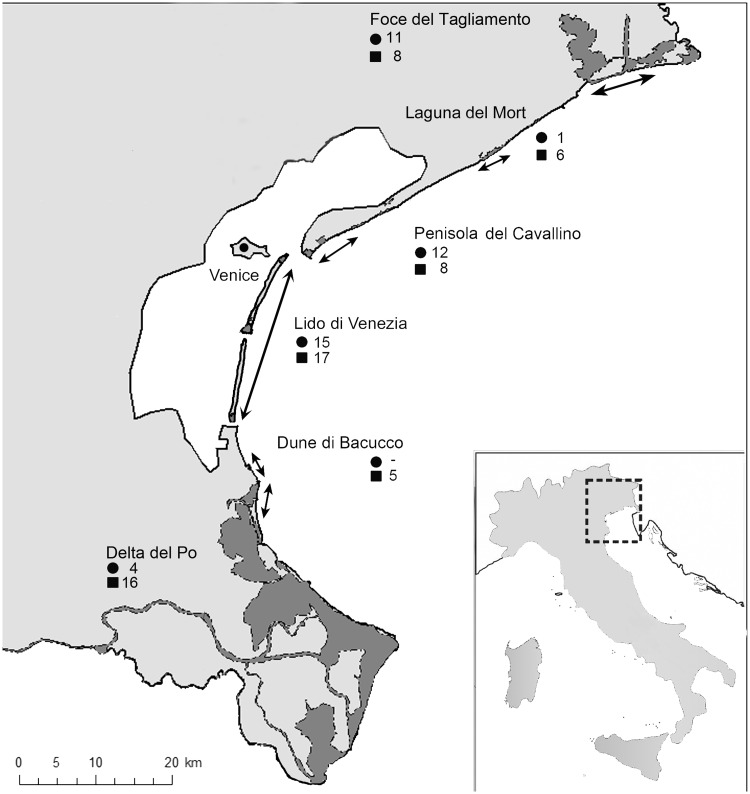
Location of the six investigated protected sites (dark grey) within the study area. The arrows indicate the extension of the coast occupied by sand dune environments, where the plot were collected. For each site, the number of plots used in the analysis is also reported; filled circle = 2000s; filled square = 2012.

The study area corresponds to the Venetian portion of the north Adriatic coast, delimited by the estuaries of the Adige and Tagliamento rivers, north-eastern Italy (Fig. 1). Sites consisted of narrow, recent dunes (Holocene), bordered by river mouths and tidal inlets, mostly fixed by docks (ARPAV 2008). Recent dunes at the sites are in contact with ancient dunes (Pleistocene), alluvial or lacustrine deposits, or run bordering the Venice Lagoon.

Sediments on the backshore and dunes are similar at all sites and are in the range of fine sand (Bezzi *et al.* 2009). Carbonate dominates the mineralogical composition of sands (especially in the northernmost area) due to the lithology of the catchment areas of corresponding rivers. Southwards a slight magmatic component arises (Zunica 1971). The predominant winds are from the northeast and east (Bezzi *et al.* 2009). Annual average wave heights are <0.50 m (Dal and Simeoni 1994) with semi-diurnal tides ranging from 1.0 m (spring) to a neap range of ∼0.20 m (Polli 1970). The combination of spring tides, winds and low atmospheric pressure can exceptionally raise sea level up to 1.60 m.

The vegetation zonation follows a typical sea-inland ecological gradient, spanning annual dominated plant communities on the strandline zone of the beach to shrubby or forest communities on the stabilized dunes (Buffa *et al.* 2012).

Until the 1950s, the Venetian coast was almost entirely fronted by dunes up to 10-m high (Bezzi and Fontolan 2003; Pignatti 2009), but few of these still survive. The coastline suffers from increasing erosion, reduction in sand supply, alteration of geomorphic processes and heavy human use, in the form of housing, resort development and road construction (Nordstrom *et al.* 2009). Summer beach tourism has become one of the region’s main sources of income and in 2011, from May to September, numbered more than 25 million visitors (Romano and Zullo 2014). In particular, trampling by beach visitors is considered one of the principal causes of degradation, affecting dune vegetation both at the species and community level (Santoro *et al.* 2012).

Despite this situation, the Venetian coastline represents one of the regional and national biodiversity hotspots, hosting many rare plant and animal species of biogeographical importance, one Protected Area (National Law 394/91), two Important Plant Areas (Blasi *et al.* 2011), and several Faunal Oases. The Natura 2000 Network includes six coastal sand dune sites (Buffa and Lasen 2010), distributed along a coastal strip of about 100 km (Fig. 1), covering a total area of about 8300 ha. These dune systems host a number of plant communities endemic to the north Adriatic coastline (Buffa *et al.* 2012; Sburlino *et al.* 2008, 2013), and are included as ‘Natural habitats of European Community interest’ in Annex I of the Habitat Directive (EEC 2013). Three of these, ‘Fixed coastal dunes with herbaceous vegetation (grey dunes)’, ‘Coastal dunes with *Juniperus* spp.’ and ‘Wooded dunes with *Pinus pinea* and/or *Pinus pinaster*’, are considered within the category of ‘priority habitats’.

### Data collection

Vegetation sampling was carried out between April and July 2012, using a stratified random sampling design on dunes that had historical vegetation survey records (Fig. 1).

The study focused on plant communities of two coastal zones: foredunes and semi-fixed or transition dunes. Foredunes (hereafter FD), which comprise embryonic and mobile dunes (habitat 2110 ‘Embryonic shifting dunes’, and 2120 ‘Shifting dunes along the shoreline with *Ammophila arenaria*—white dunes’ of the Habitat Directive 92/43/ECC, respectively). FD are the most dynamic part of the dune system and occupy the area directly behind the beach. FD are very sensitive to topographical and coastline dynamics (Bitton and Hesp 2013). They develop on poor sandy substrata, with high salinity content. Local scale sand movements cause burial and sand blasting of vegetation, and communities are characterized by low species richness and the percentage cover of vegetation is normally around 50–70% (García-Mora *et al.* 2000; Prisco *et al.* 2012). Tufted plants, such as the grasses *Elymus farctus* and *A.* *arenaria* dominate. Species with vegetative below-ground organs, as bulbs or rhizomes, proved particularly successful in withstanding sand burial (Brown and McLachlan 2002; Maun 2009). The semi-fixed or transition dunes (hereafter TD) (habitat 2130 ‘Fixed coastal dunes with herbaceous vegetation—grey dunes) occupy a zone between the mobile dunes with *A.* *arenaria*, and the more inland dune scrub and woodland habitats (Doing 1985). TD are harsh environments which favour drought tolerant plants. However, compared to the FD zone, TD are less exposed to salt winds, coastal erosion and sand burial, and so are more stable, supporting the highest number of plant species of the dry dune series (Isermann *et al.* 2010). Total percentage cover values are higher than in FD, often reaching 100%. The community is dominated by dwarf shrubs, perennial herbaceous erect leafy species, mosses and lichens (Sburlino *et al.* 2013).

The three considered habitats (2110, 2120 and 2130) are the most widespread and evenly distributed habitats in the study area (Buffa *et al.* 2012). Moreover, they have already been used as indicators of coastal dune conservation status at a landscape scale (Carboni *et al.* 2009). In addition, the presence of the habitat 2130 (grey dunes) represents one of the study area’s most interesting features. Although relatively common along the Atlantic, Baltic and North Sea coasts (Houston 2008), in Italy this habitat has been detected only along the North Adriatic coast (Prisco *et al.* 2012), where it is represented by an endemic perennial plant community (Sburlino *et al.* 2013).

All plant species were recorded and their projected cover was visually estimated by using the Braun-Blanquet seven-degree scale of abundance and dominance (Westhoff and van der Maarel 1978) in 2 × 2 m sample plots, a size commonly judged adequate in Mediterranean coastal dune ecosystems (Carboni *et al.* 2009). Altogether 60 plots were surveyed in 2012. Nomenclature and taxonomy followed Conti *et al.* (2005). Data collected in 2012 have been compared with data from previous studies carried out using the same surveying method and at the same areas in the 2000s (mostly the authors’ unpublished surveys and a small number from Gamper (2002) and Poldini *et al.* (1999). In order to obtain a homogeneous data set in terms of plot size (Haveman and Janssen 2008), only 2 × 2 m plots were selected. Overall, we obtained a matrix of 115 species × 103 plots (43 from 2000s + 60 from 2012). Plots were distributed as reported in Table 1. Plots location and number are indicated in Figure 1.

**Table 1.** List and number of plots used in the analysis. FD, embryonic and mobile dune communities; TD, transition dune communities.

Dune zone	2000s	2012
FD	7	8
	8	22
TD	28	30

### Selection of community level variables

According to the Habitat Directive, a habitat type can be considered to be at a ‘FCS’ when its ‘typical species’ are at FCS, although no clear definition of ‘typical species’ is provided (Evans and Arvela 2011). For the purposes of this study, we considered ‘typical species’ to be those that exhibited high abundances or frequencies within a vegetation type, relative to other types (Chytrý *et al.* 2002), or were structural dominants or functionally distinct elements, governing vegetation dynamics and reflecting vegetation properties such as space occupancy pattern or resistance to disturbance (De Bello *et al.* 2010; Moretti *et al.* 2009; Noss 1990).

Regarding composition, we first identified two groups of species based on species’ origin: native vs. alien or allochthonous species, the latter identified according to Celesti-Grapow *et al.* (2010). Following an approach adopted in other research (Bartha *et al.* 2008; Buffa and Villani 2012), native species were then grouped according to species’ affinity to a given habitat (ecological groups). The three resulting groups were as follows: (i) focal species, i.e. the key species pivotal to habitat structure and function, identified with reference to the list of diagnostic and characteristic species listed in Biondi *et al.* (2009), EEC (2013) and Prisco *et al.* (2012); (ii) generalist species, i.e. all native opportunistic species not specific to dune environments; and (iii) species of other habitats, i.e. all the native species that were descriptors of dune habitats other than FD and TD.

Structural features were analyzed by grouping species according to their leaf-stem architecture (growth forms), as an indicator of adaptation to both biotic and abiotic environmental conditions. Following Perez-Harguindeguy *et al.* (2013), six groups were identified: (i) erect leafy; (ii) creeping; (iii) rosette; (iv) tussocks; (v) dwarf shrubs; and (vi) shrubs and trees.

For each habitat, we calculated the following variables: (i) mean number of species per plot; (ii) mean total species cover per plot (calculated by summing the percentage cover of each species, thus obtaining a figure that can exceed 100%) (Chytrý *et al.* 2005; Del Vecchio *et al.* 2015b); (iii) mean evenness index *J* per plot, as *H’*/ln *S*, where *H’* is the Shannon diversity index and *S* the number of species; (iv) mean percent cover per plot of alien species; (v) mean percent cover per plot of each ecological group (focal species, generalist species and species of other habitats); and (vi) mean percent cover per plot of the individual growth forms.

### Data analysis

To analyze and compare species composition of FD and TD, we performed an ordination on species cover (DCA on a matrix of 103 plots × 115 species; software Juice; Tichý 2002).

To test the changes in species composition over time, we performed a Multi-Response Permutation Procedure (MRPP; PC-ORD 5.10; McCune and Mefford 2006) using the year as a grouping variable. Furthermore, we applied the Indicator Species Analysis (grouping variable = year; PC-ORD 5.10; Dufrêne and Legendre 1997) to detect the species that underwent major changes over time. This method combines information on species abundance and occurrence in a particular group, produces indicator values (IVs) for each species in each group, and tests it for statistical significance using a randomization technique. The IVs range from zero (no indication) to 100 (perfect indication).

To test the variation in the structure of vegetation over time for each dune zone (FD and TD), we ran separate null models on each of the 13 variables described above in Selection of community level variables section (species richness, species cover, evenness index, alien species, focal species, generalist species, species of other habitats and the six growth forms). We applied the Monte Carlo *F*-test for two groups (Ecosim; Gotelli and Entsminger 2004), using the year as the grouping variable (2000s and 2012; factors with two levels). The test calculates the observed *F*-value (*F*_obs_) and a simulated average *F-*value (*F*_exp_), computed on 30 000 randomly permutated matrices. This number of permutation avoids algorithm biases (Lehsten and Harmand 2006). *F*_obs_ and *F*_exp_ were then compared, calculating the probability (*P*) of the null hypothesis that *F*_obs_ was drawn at random from the distribution of the simulated *F* indexes (*F*_exp_). Non-random differences were assumed when *P*_*F*__obs ≥ __*F*__exp_ ≤ 0.05. In interpreting the *P* values we applied the Bonferroni correction for multiple comparisons to reduce type I errors, and statistical significance was consequently set at *P* < 0.003.

## Results

### Species composition: diagnostic components

**Figure 2.**
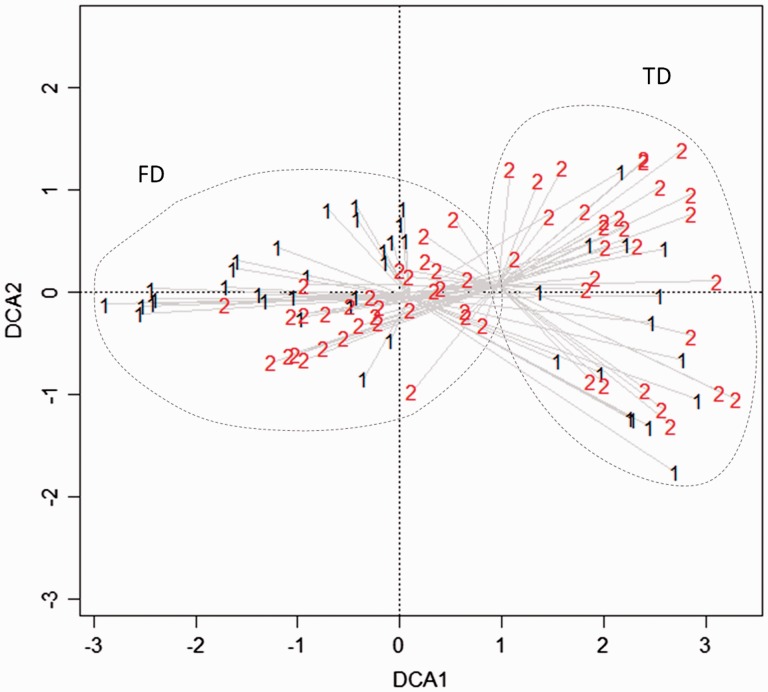
Ordination scatter diagram of sampled plots, using species as explanatory variables. Only the first two axes are represented. Number ‘1’: 2000s plots; number ‘2’: 2012 plots.

The ordination scatter diagram (Fig. 2) indicated groups of surveys according to their floristic composition. In particular, axis one shows a distinctive separation between FD and TD, reflecting the strong coastal dune zonation along the sea-inland environmental gradient.

**Figure 3.**
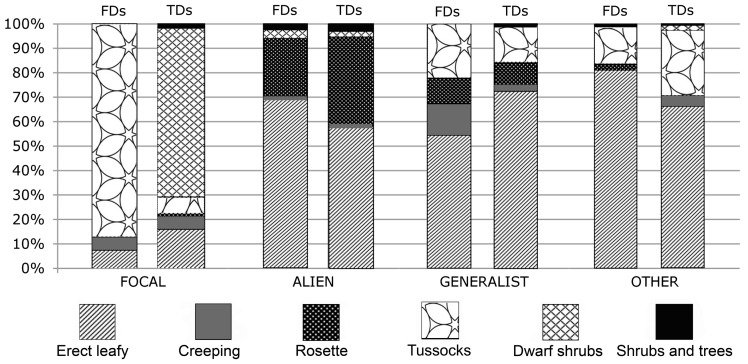
Distribution of growth forms within the selected species groups in the two zones. FD, fore dune zone; TD, transition dune zone.

The two zones demonstrated strong differences both in composition and structure, with TD showing higher mean number of species per plot and higher mean percentage cover per plot compared with FD [**see** **Supporting Information**].

Focal species were rarely shared between the two zones and only focal species of FD were recorded inland. Among the diagnostic component (focal species), those that contributed the most to distinguish the two habitats were tussocks of *A.* *arenaria* (Pearson correlation with ordination axis 1: *r* = 0.7), *E.* *farctus* (*r* = 0.3) and erect leafy species such as *Echinophora spinosa* (*r* = 0.3) in FD, while TD showed a more complex structure dominated by dwarf shrubs of *Fumana procumbens* (*r* = 0.6), *Helianthemum nummularium* ssp. *obscurum* (*r* = −0.3), *Teucrium capitatum* (*r* = −0.3), *Teucrium* *chamaedrys* (*r* = −0.3), and perennial herbs (erect leafy, rosette and tussocky plants*)* as *Petrorhagia saxifraga* (*r* = −0.5), *Koeleria macrantha* (*r*  = −0.4), *Stachys recta* (*r* = −0.3), *Silene otites* (*r* = −0.3), *Lomelosia argentea* (*r* = −0.2).

Native species of other habitats showed a similar distribution. Nitrophilous *a*nnuals typical of the upper zone of the beach (*Cakile maritima* or *Salsola kali*) colonized only the FD zone. Conversely, species of other habitats found in TD mostly came from the shrub-covered fixed dunes and for the most part were represented by woody perennials (e.g. *Helichrysum italicum*, *Asparagus acutifolius*, *Juniperus communis*) [**see** **Supporting Information**].

The group of alien species contributed to homogenize the two zones. Albeit with some exceptions, alien species colonized both zones with comparable percentage cover, irrespective of their identity, growth form and life span. Among the alien species, the most frequent and abundant growth form was that of erect leafy, e.g. *Ambrosia psilostachya*, *Cenchrus longispinus* or *Erigeron canadensis*, followed by that of rosettes, such as *Oenothera stucchii*.

### Temporal trends

**Figure 4.**
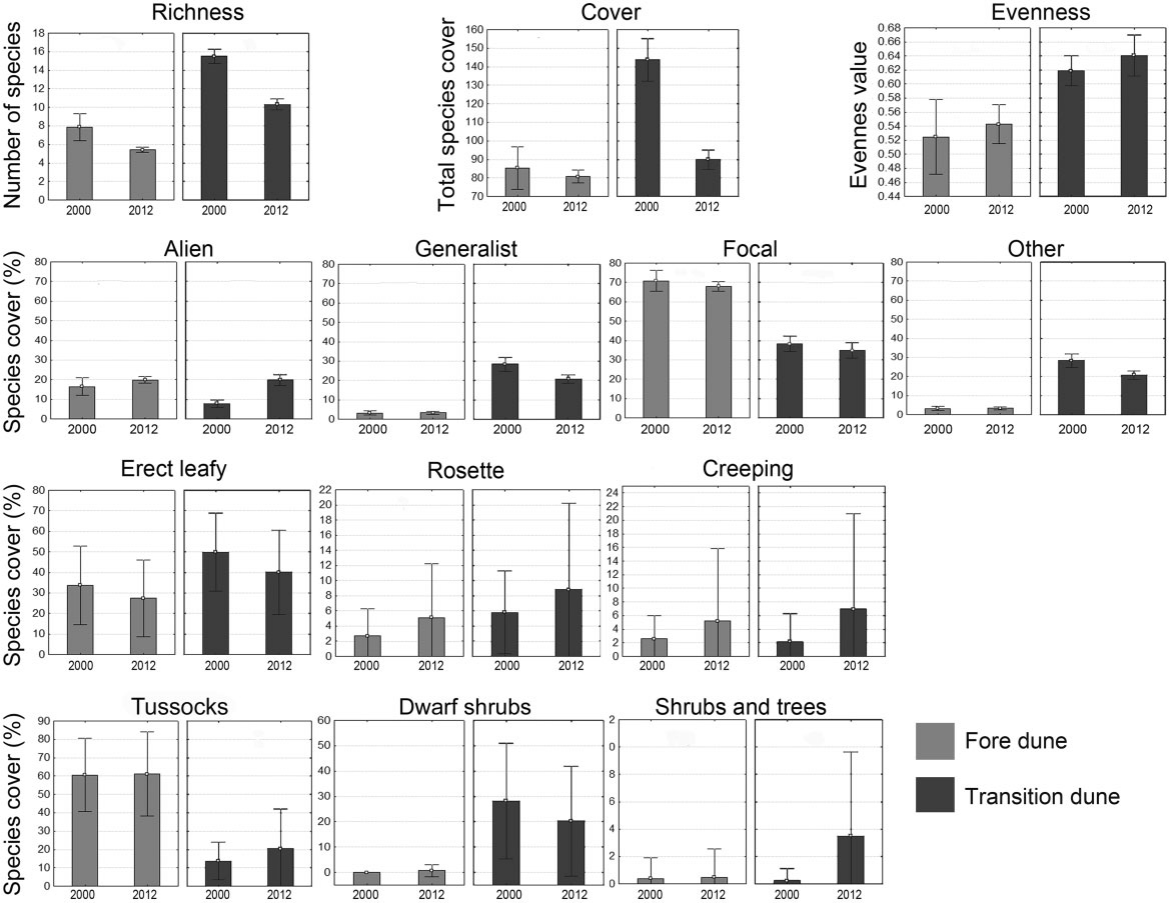
Means ± SD of the analysed fine-scale biotic variables in the FD and in the TD.

FD and TD zones showed analogous trends of variation with respect to the variables analyzed. However, only the TD zone revealed significant differences in the Monte Carlo test (Table 2 and Fig. 4). Overall, the TD zone showed a significant reduction in the mean species richness per plot and mean species cover per plot. These overall changes in community attributes reflected more fine-grained modification and turnover in species composition, ecological groups and growth forms.

**Table 2.** Results of Monte Carlo test for two groups. The critical *P*-value of 0.05 yielded a value of 0.003 according to the Bonferroni correction.

		Fore dune					Transition dune				
		Observed mean		*F-*obs	*F-*exp	*P* (O > E)	Observed mean		*F-*obs	*F-*exp	*P* (O > E)
		2000	2012				2000	2012			
**Richness**		7.9	5.2	5.46	1.04	0.0206	**15.5**	**10.5**	**27.09**	**1.02**	**0.0000**
**Cover**		85.3	76.1	0.66	1.06	0.4264	**143.8**	**89.1**	**19.60**	**1.03**	**0.0001**
Evenness		0.5	0.5	0.03	1.06	0.8563	0.6	0.7	0.68	1.03	0.4075
**Alien**		16.5	20.1	0.55	1.04	0.4625	**7.8**	**20.3**	**12.67**	**1.03**	**0.0007**
Generalist		3.2	4.1	0.18	1.02	0.6993	28.3	20.3	3.28	1.03	0.0774
Focal		70.8	64.9	0.62	1.04	0.4401	38.2	33.6	0.72	1.03	0.3967
Other		9.5	10.9	0.09	1.04	0.7712	25.7	25.2	0.01	1.04	0.9245
Erect leafy		33.7	26.9	1.43	1.06	0.2382	49.9	40.8	3.09	1.04	0.0850
Rosette		2.7	7.7	4.46	1.06	0.0452	5.8	9.1	1.89	1.04	0.1775
Creeping		2.6	1.5	0.82	1.04	0.3949	2.2	7.2	3.21	1.02	0.0762
Tussocks		60.6	62.7	0.04	1.06	0.7424	13.7	18.4	0.18	1.02	0.2573
Dwarf shrubs		0.0	0.3	1.00	1.03	0.5471	28.2	20.9	1.54	1.04	0.2168
**Shrubs and trees**		0.4	1.0	0.49	1.03	0.5930	**0.2**	**3.6**	**7.96**	**1.03**	**0.0008**

When comparing the two time steps, MRPP test revealed significant differences in species composition (*A* = 0.010; *P* < 0.001) between past and present surveys. The Indicator Species Analysis confirmed the trend, indicating that all the species that were best represented in the 2000s underwent major decline over time, as indicated by the change in their IV: *F.* *procumbens* (IV_2000s_ = 36.1 *P* = 0.001; IV_2012 _=_ _3), *E.* *spinosa* (IV_2000s_ = 28.7 *P* = 0.007; IV_2012 _=_ _4), *Scabiosa triandra* (IV_2000s_ = 24 *P* = 0.001; IV_2012 _=_ _1), *K.* *macrantha* (IV_2000s_ = 19.9; *P* = 0.004; IV_2012 _=_ _0), *Medicago marina* (IV_2000s_ = 17.8 *P* = 0.006; IV_2012 _=_ _1), *E.* *farctus* (IV_2000s_ = 19.9 *P* = 0.049; IV_2012 _=_ _4), *Medicago minima* (IV_2000s_ = 16.4 *P* = 0.008; IV_2012 _=_ _1), *S.* *otites* (IV_2000s_ = 14.0 *P* = 0.004; IV_2012 _=_ _3), *T.* *capitatum* (IV_2000s_ = 14.0 *P* = 0.004; IV_2012 _=_ _0). Although the mean cover of focal species remained more or less constant in both zones, some focal species were no longer recorded in 2012 compared with the 2000s, or showed a drop in their percentage cover. The decreasing trend was particularly evident for erect leafy species and dwarf shrubs [**see** **Supporting Information**].

In both zones, alien species showed a rising trend with respect to the past plots (Fig. 4), but only TD faced a significant increase in their mean percentage cover per plot, due to both an increased cover of some species (*A.* *psilostachya* and *O.* *stucchii*) and the arrival of new species (e.g. *Senecio inaequidens*; IV_2000s_ = 0; IV_2012 _=_ _10 *P* = 0.048) in 2012.

Growth forms showed a similar trend with respect to the past plots in both zones, but only the cover of shrubs and trees in TD showed a significant increase (Fig. 4).

## Discussion

Our study showed distinct patterns of habitat modification over time. The most evident result was an overall tendency towards a shift in the plant communities’ attributes, with lower mean species richness per plot, reduced mean species cover, increased cover of alien species and, in general, shifts in the cover of different groupings of plants, so mirroring an overall shift in both the composition and structure of the analyzed plant communities. Overall, our results are in line with other previous studies, reporting habitat degradation as a result of the increasing intensity of coast-bound tourism, excessive visitor pressure and trampling (Acosta *et al.* 2013; Davenport and Davenport 2006; Faggi and Dadon 2011; Grunewald and Schubert 2007; Provoost *et al.* 2011; Rodgers 2003; Santoro *et al.* 2012).

Given the current consensus on the positive effect of biodiversity on ecosystem functioning (Balvanera *et al.* 2006; Cardinale *et al.* 2012), species richness and loss are among the simplest and most used measures of decline in vegetation quality. Indeed, species loss under increasing land-use intensity has already been reported for different species groups (Hoffmann and Zeller 2005; Kleijn *et al.* 2009; Msuha *et al.* 2012; Verhulst *et al.* 2004). However, species richness and loss indices assign an equal weight to all species, and as such they may fail in detecting changes if coupled with the increase in other species such as alien species. Arguably, by leaving vacant niches, the disappearance of native species may contribute to making plant communities more susceptible to the invasion of alien or generalist weeds (Elton 2000; Gerhold *et al.* 2011). Hence, by overlooking the identity of species, species richness and loss indices fail in evidencing species turnover and changes in species composition resulting from some species becoming locally extinct and others entering the community. Thus, even when richness or total cover remains constant over the decades, some replacement of species can take place. Besides, not only the disappearance but also small changes or a decline in species’ abundance can lead to the disruption of a community’s structure and function, even before any characteristic component is actually lost (Keith *et al.* 2013). Ultimately, not only the total species richness but also the identity of species present, the abundance of each species, and the species’ pattern of space occupancy are crucial in determining the relationships between species diversity and ecological functions.

Species are different and do not contribute equally to ecosystem functioning (Lefcheck *et al.* 2015). Thus, the effect of species loss is likely to depend on the range of function of species in any particular community (Rosenfeld 2002). Recent research has pointed out that functional diversity, rather than the simple number of species, plays a crucial role in regulating ecosystem processes (Diaz and Cabido 2001; Hooper *et al.* 2005; Lavorel and Garnier 2002). Undoubtedly, increasing land-use intensity affects the functional diversity, and thus the stability of ecosystems and their adaptability to future changes (Cadotte *et al.* 2009; Forest *et al.* 2007; Hooper *et al.* 2005). We argue that the analysis of suitable species groups (e.g. focal species, aliens, and growth forms) is more effective in describing the relationship between disturbance patterns and biodiversity than the use of species richness per se (Carranza *et al.* 2012; Vandewalle *et al.* 2010). As for the studied coastal plant communities, focal species, which define the habitat identity, proved to be very helpful in discriminating between habitat types and zones, as they actually exhibited higher abundances or frequencies within a habitat type, relative to other habitats. Moreover, the detailed analysis of focal species abundance and turnover can be used as a short-term alert of plant community disruption, before the effects of disturbance become fully evident.

However, in coastal ecosystems, growth forms play a key role in ecosystem organization and functioning, representing essential components of vegetation identity (Maun 2004, 2009). Furthermore, being easily observable, they may provide readily discernible evidence of ongoing processes of habitat modification (Espejel *et al.* 2004; Noss 1997). In particular, when integrated with compositional indicators, they can help evaluate changes and trends caused by disturbance and anthropogenic stresses (Espejel *et al.* 2004).

Although spatially close, investigated systems showed some distinctive features in terms of composition, structure and pattern of spatial occupancy, and a high level of distinctiveness. These peculiar features determined the different behaviour which the two zones exhibited in response to pressure exerted along the temporal sequence. Indeed, our results showed similar trends in the selected variables comparing former vs. current situation in both zones, but with different statistical significance. This suggests that the ability of the selected variables to identify changes is related to the structural complexity of the vegetation type under evaluation.

Several authors agree in assuming that the alteration of plant communities and, eventually, the disappearance of the most sensitive are generally linked to the alteration of the morphology of dune systems (Acosta *et al.* 2007; Nordstrom *et al.* 2007). In the Mediterranean coasts, especially tourism, and associated trampling and beach cleaning operations, appears to have a detrimental impact on sand dunes habitats (Ciccarelli 2014; Farris *et al.* 2013; Pinna *et al.* 2015; Santoro *et al.* 2012).

According to Cole (1995), the variability of the response to human disturbance, e.g. trampling, was mainly due to plant morphological characteristics, namely growth forms, than to site characteristics such as topography. Overall, tolerance, i.e. the ability of plants and plant communities to withstand a cycle of disturbance and recover, seemed primarily a function of stature, erectness and growth form. The most tolerant plants were in order tufted graminoids, rosette and creeping plants, and woody plants (shrubs and trees), while the least tolerant were the dwarf shrubs. As resistance was mainly a function of vegetation stature and erectness, erect leafy plants turned out to be the least resistant growth form.

Thus, given the intrinsic low species richness and low percentage cover (Del Vecchio *et al.* 2015b; Prisco *et al.* 2012) coupled with the leaf-stem architecture of their resident focal species, mainly tufted species, FD appear to be more capable of withstanding and recovering after disturbance events (Lucas and Carter 2013) and changes may be expected to occur more slowly compared with TD. Conversely, TD which are dominated by dwarf shrubs (Sburlino *et al.* 2013), are amongst the sand dunes communities most sensitive to trampling.

The group of alien species partially represents an exception to the rule, demonstrating an increasing trend irrespective of species’ identity and growth form. In fact, as well as a trampling tolerant rosette species (*O.* *stucchii*), erect leafy species (e.g. *A.* *psilostachya* or *C.* *longispinus*), dwarf shrubs (e.g. *S.* *inaequidens*) and shrubs and trees (e.g. *Amorpha fruticosa*, *Elaeagnus angustifolia*) also showed stable or increasing mean percentage cover. Indeed, increasing alien population growth and dispersal have been proved to be favoured by widespread anthropogenic environmental alterations which create new, suitable habitats and ensure human-assisted dispersal, reducing the distinctiveness of plant communities (Del Vecchio *et al.* 2015a; Pyšek and Hulme 2005). Thus, also the proportion and cover of alien species can provide diagnostic information, which would be disregarded when considering diversity pattern alone.

## Conclusions

Our study may help to highlight some challenging points. First, although species richness is traditionally the most widely considered component of biodiversity in conservation planning, our study emphasized that species richness on its own cannot explain all patterns of biodiversity (Bartha *et al.* 2008; Van Meerbeek *et al.* 2014). The analysis of community level variables, in particular ecological groups such as native focal species and aliens, and growth forms, seems to be a practical tool for assessing and monitoring vegetation quality in coastal environments. In this context, the most interesting take home message stemming from the current study is that the analysis of suitable species groups may provide an instrument for the assessment of decline that can be applied to other coastal systems. Moreover, the plant community level proved to be effective in detecting the fine-scale habitat heterogeneity and the diversity of species assemblages in sandy coastal systems, which, in many cases, are addressed at a too coarse scale and disappear in large scale management plans.

Second, at the local scale, our study underlines the need for monitoring and active management of these environments particularly where TD are concerned. Across Europe transition and fixed dunes are the most threatened and exploited part of the dune system (Genovesi *et al.* 2014; Houston 2008). Although plant communities are expected to possess a certain capability of resisting external fluctuations, that is a ‘biological inertia’ (Gorham 1957), TD have shown changes in species’ composition and abundance over a short period of time, a process that should not be underestimated. In fact, for vegetation types, disappearance could be incremental, due to gradual changes in their characteristic features; as such they may not become extinct, but rather turn into a new type with a new species combination and potentially new functions (Hobbs *et al.* 2006).

Finally and on a broader scale our results contribute to underlining a noteworthy aspect concerning the Natura 2000 network, which is considered one of the most important and largest conservation networks worldwide (Hochkirch *et al.* 2013; Maiorano *et al.* 2007) and one of the most important tools that could allow to improve the existing networks of conservation areas and to meet the target of halting biodiversity loss. One of the principal debates in the field of conservation ecology is the monitoring of results obtained by conservation targets and the evaluation of their efficacy, i.e. the ability to achieve conservation targets, of existing protected areas (Jackson *et al.* 2009; Vellak *et al.* 2009). As required by the EC Habitats Directive, Natura 2000 sites in the North-Adriatic dune system were selected in order to ensure the long-term persistence of its native biodiversity, i.e. species and habitats of European interest. Despite this, since their formal establishment as Natura 2000 sites no official management plans have been approved, competition over land-use allocation still remains a problem and most dune habitats, as many other habitat types (Bagella *et al.* 2013), are still under heavy pressure. According to data reported by the Italian Ministry of the Environment and Protection of Land and Sea (Genovesi *et al.* 2014), 67.6% of habitats are currently characterized by inadequate or very bad conservation status, percentage that rises to 86.7% for coastal habitats, which are threaten mostly by urban sprawl, linked to the explosion of mass tourism.

Although recreational tourism is economically stimulating in terms of income, employment and development, it is also inevitably associated with a heavy impact. Intense tourism pressure coupled with a general lack of ecological consciousness of the value of these ecosystems may ultimately compromise not only the natural value and the ecological functionality of these systems, but also the quality of the recreational experience itself. Thus, in contexts where high-value natural ecosystems and socio-economic interests coexist, halting biodiversity loss requires substantial societal and political consensus as well as the determination to implement conservation strategies at all administrative levels, from national authorities to regional administrations, and local stakeholders.

## Sources of Funding

This study was supported by the internal research grant for Department of Environmental Science, Informatics and Statistics, Ca’ Foscari University of Venice, Italy.

## Conflicts of Interest

None declared

## Supplementary Material

Supplementary Data

## Supporting Information

The following **[Supporting Information**] is available in the online version of this article –

**File 1.** Table. List of species surveyed in the FD and TD zones in 2000 and 2012. Species with frequency values <1% are not displayed in the table. Ecological group symbols mean as follows: F-focal; A-aliens; G-generalist; OH-other habitats. Growth forms are indicated in number as follows: 1-erect leafy; 2-creeping; 3-rosette; 4-tussock; 5-dwarf shrubs; 6-shrubs and trees. Cumulative species number refers to the overall pool of species surveyed in both time steps. Braun-Blanquet rank cover scale was converted in percentage as follows: 5 = 87.5%; 4 = 62.5%; 3 = 37.5%; 2 = 15%; 1 = 2.5%;  + =1%; r = 0.1% (van der Maarel 1979).
