# Supplementary material for: The use of plant community attributes to detect habitat quality in coastal environments
Source: AoB Plants. 2016 Jul 11;8:plw040. doi: 10.1093/aobpla/plw040 (PMC4940507; doi:10.1093/aobpla/plw040)
Supplement: Supplementary Data [file supp_8_plw040_index.html]

The use of plant community attributes to detect habitat quality in coastal environments — Supplementary Data 

# The use of plant community attributes to detect habitat quality in coastal environments

## Supplementary Data

files

- Supplementary Data - doc file
