# Supplementary material for: The use of plant community attributes to detect habitat quality in coastal environments
Source: AoB Plants. 2016 Jul 11;8:plw040. doi: 10.1093/aobpla/plw040 (PMC4940507; doi:10.1093/aobpla/plw040)
Supplement: Supplementary Data [file supp_plw040_aobplants-15196-s02.doc]

|  | FDs | | | | TDs | | | |  |  |
| --- | --- | --- | --- | --- | --- | --- | --- | --- | --- | --- |
|  | **2000** | | **2012** | | **2000** | | **2012** | |  |  |
| Number of surveys | 15 | | 30 | | 28 | | 30 | |  |  |
| Cumulative species number | 49 | | | | 98 | | | |  |  |
|  | Mean  Cover (%) | SD | Mean  Cover (%) | SD | Mean  Cover (%) | SD | Mean  Cover (%) | SD | Ecological groups | Growth form |
| Mean species richness per plot | 7.87 | 5.58 | 5.39 | 2.21 | 15.46 | 4.17 | 10.20 | 3.01 |
| Mean total species cover per plot | 85.33 | 43.84 | 81.20 | 28.72 | 143.61 | 60.69 | 86.47 | 30.20 |
|  |  |  |  |  |  |  |  |  |  |  |
| Euphorbia paralias | **0.67** | 1.76 | **0.60** | 2.57 | **-** | **-** | **-** | **-** | F-FDs | 1 |
| Echinophora spinosa | **7.73** | 9.82 | **1.30** | 3.88 | **0.71** | 1.78 | **0.70** | 2.81 | F-FDs | 1 |
| Calystegia soldanella | **0.20** | 0.77 | **2.93** | 6.52 | **0.36** | 1.31 | **0.70** | 1.64 | F-FDs | 2 |
| Medicago marina | **1.47** | 1.96 | **0.29** | 1.87 | **0.71** | 1.78 | **0.60** | 2.77 | F-FDs | 2 |
| Ammophila arenaria | **32.93** | 32.11 | **46.37** | 34.69 | **0.29** | 1.08 | **0.17** | 0.91 | F-FDs | 4 |
| Cyperus capitatus | **1.20** | 3.90 | **-** | - | **1.36** | 3.23 | **5.93** | 15.67 | F-FDs | 4 |
| Elymus farctus | **14.13** | 13.94 | **4.84** | 8.39 | **0.18** | 0.94 | **0.17** | 0.91 | F-FDs | 4 |
| Centaurea tommasinii | **-** | **-** | **-** | **-** | **0.18** | 0.94 | **-** | - | F-TDs | 1 |
| Stachys recta | **-** | **-** | **-** | **-** | **0.71** | 1.78 | **0.17** | 0.91 | F-TDs | 1 |
| Lomelosia argentea | **0.20** | 0.77 | **-** | - | **5.89** | 10.70 | **6.00** | 10.27 | F-TDs | 1 |
| Silene conica | **-** | - | **0.07** | 0.59 | **0.82** | 1.83 | **0.33** | 1.27 | F-TDs | 1 |
| Thymus x carstiensis | **-** | **-** | **-** | **-** | **0.71** | 2.95 | **4.40** | 11.72 | F-TDs | 2 |
| Silene otites | **-** | **-** | **-** | **-** | **0.93** | 1.86 | **-** | - | F-TDs | 3 |
| Koeleria macrantha | **-** | **-** | **-** | **-** | **3.00** | 5.27 | **0.67** | 2.86 | F-TDs | 4 |
| Petrorhagia saxifraga | **-** | **-** | **-** | **-** | **1.79** | 2.44 | **1.10** | 2.06 | F-TDs | 4 |
| Fumana procumbens | **-** | **-** | **-** | **-** | **34.07** | 30.35 | **9.93** | 18.61 | F-TDs | 5 |
| Helianthemum nummularium subsp obscurum | **-** | **-** | **-** | **-** | **1.21** | 1.73 | **2.17** | 5.20 | F-TDs | 5 |
| Teucrium capitatum | **-** | **-** | **-** | **-** | **8.29** | 18.61 | **-** | - | F-TDs | 5 |
| Teucrium chamaedrys | **-** | **-** | **-** | **-** | **2.82** | 7.97 | **0.93** | 2.98 | F-TDs | 5 |
| Teucrium montanum | **-** | **-** | **-** | **-** | **0.11** | 0.57 | **0.50** | 2.74 | F-TDs | 5 |
| Ambrosia psylostachya | **0.53** | 1.46 | **3.86** | 6.91 | **3.96** | 5.52 | **7.13** | 8.21 | A | 1 |
| Cenchrus longispinus | **2.20** | 4.11 | **1.37** | 3.66 | **-** | - | **1.50** | 3.26 | A | 1 |
| Conyza albida | **-** | - | **0.04** | 0.36 | **-** | **-** | **-** | **-** | A | 1 |
| Erigeron canadensis | **1.33** | 3.99 | **0.20** | 0.84 | **0.36** | 1.31 | **0.53** | 1.43 | A | 1 |
| Xanthium orientale ssp. italicum | **4.33** | 3.42 | **4.80** | 5.43 | **-** | - | **0.33** | 1.27 | A | 1 |
| Cuscuta cesatiana | **0.67** | 1.76 | **-** | - | **-** | - | **0.17** | 0.91 | A | 2 |
| Oenothera stucchii | **2.47** | 3.96 | **3.71** | 5.20 | **3.04** | 3.26 | **5.53** | 8.73 | A | 3 |
| Senecio inaequidens | **-** | - | **0.70** | 2.65 | **-** | - | **0.53** | 1.43 | A | 5 |
| Amorpha fruticosa | **0.33** | 1.29 | **0.30** | 1.11 | **-** | **-** | **-** | **-** | A | 6 |
| Elaeagnus angustifolia | **-** | **-** | **-** | **-** | **-** | - | **0.17** | 0.91 | A | 6 |
| Tamarix gallica | **0.20** | 0.77 | **-** | - | **-** | - | **0.60** | 2.77 | A | 6 |
| Anisantha diandra | **-** | **-** | **-** | **-** | **0.57** | 1.48 | **-** | - | G | 1 |
| Arenaria leptoclados | **-** | **-** | **-** | **-** | **0.46** | 1.40 | **-** | - | G | 1 |
| Aristolochia clematitis | **-** | **-** | **-** | **-** | **0.54** | 1.57 | **-** | - | G | 1 |
| Asperula cynanchica | **-** | **-** | **-** | **-** | **0.36** | 1.31 | **0.50** | 2.74 | G | 1 |
| Bromus hordeaceus | **-** | **-** | **-** | **-** | **0.54** | 1.57 | **-** | - | G | 1 |
| Catapodium rigidum | **-** | **-** | **-** | **-** | **1.00** | 1.98 | **-** | - | G | 1 |
| Cerastium semidecandrum | **-** | - | **0.07** | 0.59 | **3.61** | 1.91 | **1.30** | 2.09 | G | 1 |
| Crepis vesicaria | **-** | **-** | **-** | **-** | **1.07** | 3.15 | **-** | - | G | 1 |
| Diplotaxis tenuifolia | **-** | **-** | **-** | **-** | **1.79** | 2.44 | **-** | - | G | 1 |
| Equisetum ramosissimum | **-** | **-** | **-** | **-** | **1.50** | 2.13 | **1.17** | 3.87 | G | 1 |
| Geranium rotundifolium | **-** | **-** | **-** | **-** | **0.36** | 1.31 | **-** | - | G | 1 |
| Lagurus ovatus | **-** | - | **0.59** | 2.13 | **3.71** | 7.52 | **2.50** | 2.33 | G | 1 |
| Medicago minima | **-** | - | **0.04** | 0.36 | **2.07** | 4.14 | **0.27** | 1.05 | G | 1 |
| Phleum arenarium | **0.20** | 0.77 | **0.37** | 1.24 | **3.18** | 2.31 | **3.27** | 3.87 | G | 1 |
| Silene canescens | **0.87** | 1.85 | **0.07** | 0.59 | **-** | - | **0.33** | 1.27 | G | 1 |
| Silene vulgaris | **1.20** | 2.11 | **0.09** | 0.50 | **2.68** | 2.31 | **2.37** | 2.46 | G | 1 |
| Veronica arvensis | **-** | **-** | **-** | **-** | **0.54** | 1.57 | **-** | - | G | 1 |
| Vicia villosa | **-** | **-** | **-** | **-** | **0.18** | 0.94 | **0.17** | 0.91 | G | 1 |
| Cynodon dactylon | **0.40** | 1.06 | **0.33** | 1.90 | **1.00** | 1.98 | **0.17** | 0.91 | G | 2 |
| Erophila verna | **-** | **-** | **-** | **-** | **-** | - | **0.33** | 1.27 | G | 3 |
| Hypochoeris radicata | **-** | - | **0.40** | 1.32 | **1.54** | 2.30 | **1.07** | 1.87 | G | 3 |
| Plantago lanceolata | **-** | **-** | **-** | **-** | **1.36** | 3.23 | **0.43** | 1.36 | G | 3 |
| Dactylis glomerata | **-** | **-** | **-** | **-** | **0.89** | 1.95 | **0.17** | 0.91 | G | 4 |
| Vulpia fasciculata | **-** | - | **0.84** | 1.68 | **3.25** | 4.58 | **3.43** | 3.83 | G | 4 |
| Rubus ulmifolius | **-** | **-** | **-** | **-** | **-** | - | **0.37** | 1.16 | G | 6 |
| Allium sphaerocephalon | **-** | **-** | **-** | **-** | **1.43** | 2.30 | **-** | - | OH | 1 |
| Allium vineale | **-** | **-** | **-** | **-** | **0.36** | 1.31 | **-** | - | OH | 1 |
| Apocynum venetum | **1.20** | 3.90 | **0.07** | 0.59 | **1.46** | 7.18 | **2.93** | 16.07 | OH | 1 |
| Cakile maritima | **1.87** | 2.42 | **3.53** | 5.72 | **-** | **-** | **-** | **-** | OH | 1 |
| Carex liparocarpos | **-** | **-** | **-** | **-** | **5.36** | 10.01 | **1.53** | 2.01 | OH | 1 |
| Centaurium erythraea | **-** | **-** | **-** | **-** | **0.36** | 1.31 | **0.17** | 0.91 | OH | 1 |
| Centaurium pulchellum | **-** | **-** | **-** | **-** | **0.18** | 0.94 | **0.17** | 0.91 | OH | 1 |
| Eryngium maritimum | **1.33** | 2.29 | **1.03** | 3.19 | **-** | - | **0.17** | 0.91 | OH | 1 |
| Globularia bisnagarica | **-** | **-** | **-** | **-** | **0.46** | 1.40 | **-** | - | OH | 1 |
| Hieracium piloselloides | **-** | - | **0.07** | 0.59 | **0.36** | 1.31 | **0.50** | 1.53 | OH | 1 |
| Polygonatum odoratum | **-** | **-** | **-** | **-** | **0.71** | 2.95 | **-** | - | OH | 1 |
| Salsola kali | **1.27** | 1.94 | **1.07** | 1.94 | **-** | **-** | **-** | **-** | OH | 1 |
| Sanguisorba minor | **0.20** | 0.77 | **-** | - | **10.93** | 21.89 | **2.10** | 4.05 | OH | 1 |
| Scabiosa columbaria | **-** | **-** | **-** | **-** | **1.25** | 2.20 | **-** | - | OH | 1 |
| Scabiosa triandra | **-** | **-** | **-** | **-** | **2.07** | 2.46 | **0.43** | 1.36 | OH | 1 |
| Silene italica | **-** | **-** | **-** | **-** | **1.00** | 1.98 | **0.20** | 0.76 | OH | 1 |
| Thymelaea passerina | **-** | **-** | **-** | **-** | **4.68** | 17.85 | **-** | - | OH | 1 |
| Leontodon hispidus | **0.53** | 1.46 | **-** | - | **-** | - | **0.17** | 0.91 | OH | 3 |
| Bothriochloa ischaemum | **-** | **-** | **-** | **-** | **0.36** | 1.31 | **-** | - | OH | 4 |
| Bromus erectus | **-** | **-** | **-** | **-** | **0.36** | 1.31 | **-** | - | OH | 4 |
| Chrysopogon gryllus | **-** | **-** | **-** | **-** | **0.54** | 1.57 | **-** | - | OH | 4 |
| Elytrigia atherica | **0.53** | 1.46 | **0.11** | 0.69 | **0.29** | 1.08 | **-** | - | OH | 4 |
| Elytrigia repens | **-** | - | **0.19** | 0.90 | **-** | **-** | **-** | **-** | OH | 4 |
| Holoschoenus romanus | **0.20** | 0.77 | **-** | - | **-** | - | **0.10** | 0.55 | OH | 4 |
| Poa bulbosa | **-** | **-** | **-** | **-** | **4.14** | 10.11 | **1.50** | 4.58 | OH | 4 |
| Schoenus nigricans | **-** | **-** | **-** | **-** | **0.18** | 0.94 | **0.17** | 0.91 | OH | 4 |
| Stipa veneta | **-** | **-** | **-** | **-** | **0.64** | 1.64 | **-** | - | OH | 4 |
| Helichrysum italicum | **-** | - | **0.04** | 0.36 | **-** | - | **4.57** | 8.70 | OH | 5 |
| Asparagus acutifolius | **-** | **-** | **-** | **-** | **-** | - | **0.60** | 1.59 | OH | 5 |
| Osyris alba | **-** | **-** | **-** | **-** | **0.93** | 3.01 | **0.17** | 0.91 | OH | 5 |
| Juniperus communis | **-** | **-** | **-** | **-** | **-** | - | **0.77** | 2.88 | OH | 6 |
| Pinus pinea | **-** | **-** | **-** | **-** | **-** | - | **0.50** | 1.53 | OH | 6 |
| Quercus ilex | **-** | **-** | **-** | **-** | **0.36** | 1.31 | **-** | - | OH | 6 |
| Rubia peregrina | **-** | **-** | **-** | **-** | **-** | - | **0.27** | 1.05 | OH | 6 |

**Supporting Information**: List of species surveyed in the FD and TD sectors in 2000 and 2012. Species with frequency values < 1% are not displayed. Ecological group symbols mean as follows: F-focal; A-aliens; G-generalist; OH-other habitats. Growth forms are indicated in number as follows: 1-erect leafy; 2-creeping; 3-rosette; 4-tussock; 5-dwarf shrubs; 6-shrubs and trees. Cumulative species number refers to the overall pool of species surveyed in both time steps. Braun-Blanquet rank cover scale was converted in percentage as follows: 5=87.5%; 4=62.5%; 3=37.5%; 2=15%; 1=2.5%; +=1%; r=0.1% (van der Maarel 1979).
